# An unusual suite of sexual characters in three new species of *Hymenorus* (Coleoptera, Tenebrionidae, Alleculinae) from Guatemala and Mexico

**DOI:** 10.3897/zookeys.415.6662

**Published:** 2014-06-12

**Authors:** J. M. Campbell

**Affiliations:** 1 Canadian National Collection of Insects, Arachnids, and Nematodes; Agriculture and Agri-Food Canada; Ottawa, Ontario, Canada, K1A 0C6

**Keywords:** Coleoptera, Tenebrionidae, Alleculinae, *Hymenophorus*, *Hymenorus*, Guatemala, Chiapas, taxonomy, new species

## Abstract

Two species, *Hymenorus bifurcatus*, and *H. excavatus* are described as new from Guatemala and the new species *H. balli* from both the state of Chiapas in southern Mexico and Guatemala. These three species are unique among the species of *Hymenorus* Mulsant, 1851 in the unusual and highly modified fifth ventrites of the male and the modified shape of the female ninth tergites. The unusual sexual characters of the males and females are illustrated with photographs. The usage of the generic names *Hymenorus* Mulsant versus *Hymenophorus* Mulsant is discussed.

## Introduction

The genus *Hymenorus* Mulsant 1851 is the largest, but least studied genus of Alleculinae from North and Central America. The species of Mexico and Central America were studied by Champion (1888, 1893) who included 39 species of which all but two were described as new. Fall (1931) reviewed the species of Canada, Baja California and the United States including 100 species; Marshall (1970) transferred two species from the Southwestern United States to the genus *Alethia* Champion & Campbell described one new species from California (1982), two species from Panama (1962), and 16 species from the West Indies (1971). These are the only recent studies of the New World species of the genus. I am currently revising the species of Alleculinae of Costa Rica which, when published, will add an additional eight species to the Central American fauna. In going through my collection for this study, three highly unusual species were discovered from Guatemala and Chiapas in southern Mexico. None of these resemble any of the species previously known from Central America or Mexico.

Species of *Hymenorus* may be readily distinguished from those of other genera of Alleculinae occurring in Mexico and Central America by the generic key provided by Champion (1888) in the Biologia Centrali-Americana. In North and Central America the genus has never been adequately described. Instead, any species of Alleculinae with unusual or unique characters have been removed to other genera. The remaining species are left in *Hymenorus*. In southern Mexico and Guatemala any small (less than 10 mm), light to dark brown, pubescent Alleculinae with at least some of the tarsal segments lobed that have the apical segment of the maxillary palpus securiform and the antennae moderately elongate (antennomeres four through ten from just over one to approximately two times longer than wide) have been assigned to *Hymenorus*.

## Methods

All measurements were made with an ocular micrometer mounted in a Leitz stereoscopic microscope. Measurements were made of the overall length from the anterior margin of the labrum to the apex of the elytra; the ocular index (OI) of both males and females (the distance between the eyes dorsally divided by the greatest distance across the eyes multiplied by 100); and the lengths of the third and fourth antennomeres and the length and width of the tenth antennomere. Measurements of the tenth antennomere are used for comparison because their length is less variable than those of the ultimate segment. The pronotal index (PI) is a measurement of the length of the pronotum divided by the greatest width of the pronotum multiplied by 100. The photographs were made with a Leica Digital DC500 Imaging Workstation using Zerene Stacker software and retouched with Adobe Photoshop software.

The terminology used in this paper is the same as that recommended by Lawrence et al (2010). All material included in this paper were collected by the author except for the long series of *Hymenorus balli* from Chiapas collected by George Ball and two specimens of *Hymenorus excavatus* collected by WB. Warner (WBWC). All holotypes are deposited in the Canadian National Collection of Insects, Ottawa (CNCI). Paratypes are deposited in the CNCI, my personal collection, JM Campbell, Ottawa, Canada (JMCC) and the WB Warner collection, Chandler, Arizona, USA (WBWC).

## Systematics

Usage of the generic name *Hymenorus* Mulsant and the name *Hymenophorus* Mulsant has been confused. Mulsant (1851) described *Hymenophorus* with the type species the new species *Hymenophorus doublieri*. In the emendanda section of a later paper (1852, p. 188) Mulsant changed the name to *Hymenorus* based on his belief that the name *Hymenophorus* was a junior homonym of *Hymenophora* Laporte (1843) [Hemiptera]. Mulsant (1856b, p. 20) later added a second new species to the genus, *Hymenorus rugicollis*. Subsequent papers, both from the Nearctic and Palearctic Regions have consistently used the name *Hymenorus* until recent papers by Novák (2006, 2007) and the publication of the catalogue of the Palaearctic Coleoptera (Novák and Petterrsson 2008). In recent checklists or catalogues of regions of the Palaearctic fauna, following the publication of this catalogue, the name *Hymenopohorus* has been adopted. Nearctic workers have used the name *Hymenorus* consistently since the genus was first recorded from North America (LeConte 1866) and the name *Hymenorus* has continued to be used by North American workers. The type species of both names is *Hymenophorus doublieri* Mulsant, 1851.

In this paper I have followed the usage adopted by Bouyon (2011) who follows the International Code of Zoolgical Nomenclature (ICZ, 1999, Article 35.2.3.1) in recognizing the generic name *Hymenorus* as the valid name. Because the emendation of *Hymenophorus* to *Hymenorus* is in prevailing usage and attributed to the original author and date, it is deemed to be a justified emendation (ICZ, 1999 Article 33.2.3.1) and the name thus corrected retains the authorship and date of the original spelling (ICZ, 1999, Article 33.2.2). *Hymenorus* continues to be consistently used in all recent publications of the New World species of the genus.

### Hymenorus

Mulsant, 1851

http://species-id.net/wiki/Hymenorus

Hymenophorus Mulsant, 1851, p. 201 [1852a, p. 68]; type species *Hymenophorus doublieri* Mulsant, 1851, by monotypy. Novák 2006, p. 317; Novák 2007; Novák and Petterrsson 2008, p. 322.
Hymenorus Mulsant, 1852, p. 188 [emendation]; Mulsant (1856a: 17, 33); Jacquelin du Val (1861: 344, 356); LeConte (1866: 137); LeConte and Horn (1883: 390); Champion (1888: 386, 424); Seidlitz (1896: 49); Casey (1891: 72, 83); Blatchley (1910: 1271, 1273); Reitter (1911: 351, 352); Fall (1931: 161); Chagnon and Robert (1962: 325); Campbell (1962:9 2); Arnett (1962: 703); Hatch (1965: 183); Campbell (1971: 68); Campbell (1982: 31; Campbell (1984: 296); Downie and Arnett (1996: 1099); Aalbu et al. (2002, 480, 499); Steiner (2004: 739); Althoff et al. (2005: 905); Bouyon (2011: 191); Kanda (2013: 587).

#### Description.

A full description of the New World species of *Hymenorus* is not possible at this time pending modern revisions of the more than 170 North and Central American species of the genus. However, the following brief description will readily distinguish these three species from all other New World species of *Hymenorus*.

**Figure 1–6.**
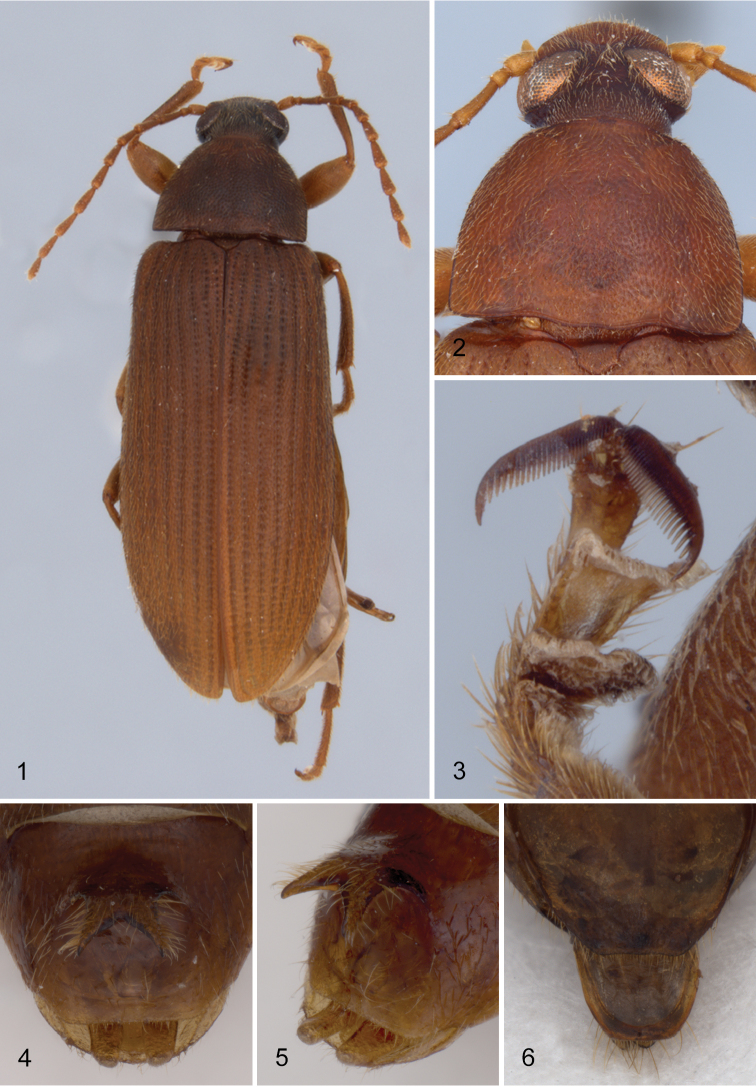
**1** dorsal habitus of *Hymenorus balli* **2** head and pronotum of *Hymenorus excavatus* **3** protarsal claws of *Hymenorus excavatus* **4** and **5** male fifth ventrite of *Hymenorus balli* **4** ventral view **5** lateral oblique view **6** dorsal view of female ninth tergite of *Hymenorus balli*.

Body narrowly elongate-oval (Fig. 1); length 7.5–10.0 mm. Eyes large, moderately separated dorsally; OI of males varying from 18 to 27, females slightly more widely separated, OI varying from 18–33. Antennae narrowly elongate, antennomeres four through ten narrowly elongate, approximately two times longer than wide. Pronotum (Fig. 2) wider than long, width at base slightly narrower than width of base of elytra; PI ranging from 62 to 78; disc with fine, dense microsculpture between punctures; punctures coarse, dense, narrowly separated, evenly distributed over disc; each puncture obliquely impressed. Metaventrite moderately densely punctate medially, punctures becoming sparser laterally; without median patches of dense, elongate setae. Like all *Hymenorus* species, the third and fourth segments of the pro- and mesotarsi and the penultimate segment of the metatarsi have a distinct membranous lobe on the ventral margins.

**Figure 7–12.**
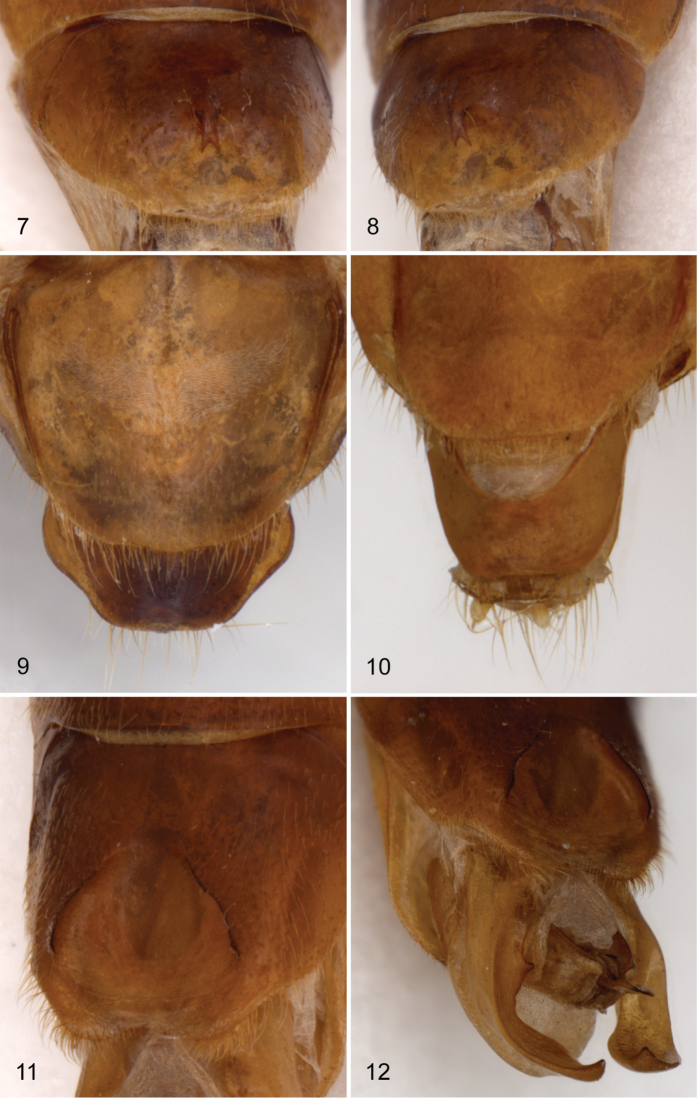
**7** and **8** male fifth ventrite of *Hymenorus bifurcatus* **7** ventral view **8** lateral oblique view **9** dorsal view of female ninth tergite of *Hymenorus bifurcatus* **10** dorsal view of female ninth tergite of *Hymenorus bifurcatus* **11** and **12** male fifth ventrite of H. excavatus **11** ventral view **12** lateral oblique view.

Male. *Hymenorus excavatus* and *Hymenorus bifurcatus* have the second segment of the anterior tarsus with a small, rudimentary lobe and a densely pubescent pad on the venter of the basal segment; only the third and fourth segments of the protarsi are lobed in *Hymenorus balli*. The anterior tarsal claws of the three species each have at least 20 teeth (Fig. 3). The fifth abdominal ventrite is highly modified, in one species (*Hymenorus excavatus*) (Figs 10, 11), the ventrite is deeply, triangularly excavate from the apical margin to the anterior third; in *Hymenorus balli* (Figs 4–5) and *Hymenorus bifurcatus* (Figs 7–8) the ventrites have a distinct, bifurcate process projecting ventrally from the middle of the disc. Lobes of eighth sternite of each species are highly modified (Figs 19–21), unlike any other species of the genus.

Female. The ninth tergite (Figs 6, 9, 12) of each species is highly modified and completely unlike any other known species of the genus. In most species of the genus the apical margin of the tergite is evenly convex and the length of the tergite varies from short to elongate.

#### Remarks.

I have not provided a key to distinguish these species. Other than sexual characters they are very similar. The modifications of the male fifth abdominal ventrite and lobes of the eighth sternite and the shape of the female ninth tergite will readily distinguish the species. In most series a few of the sexual characters are readily visible without dissections.

### Hymenorus balli sp. n.

http://zoobank.org/29D2A338-1174-4BD0-9152-E8D0ADF06A1F

http://species-id.net/wiki/Hymenorus_balli

**Figure 13–18.**
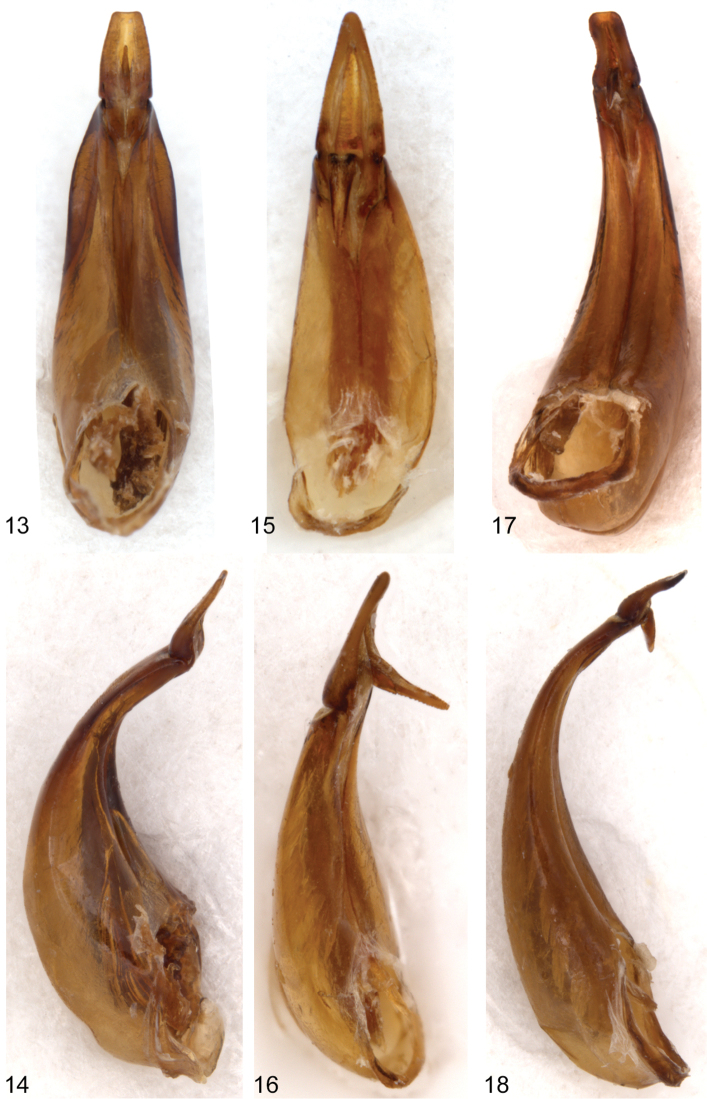
**13–14** ventral (left) and lateral (right) views of aedeagus of *Hymenorus balli* **15–16** ventral (left) and lateral (right) views of aedeagus of *Hymenorus bifurcatus* **17–18** ventral (left) and lateral (right) views of aedeagus of *Hymenorus excavatus*.

**Figure 19–21.**
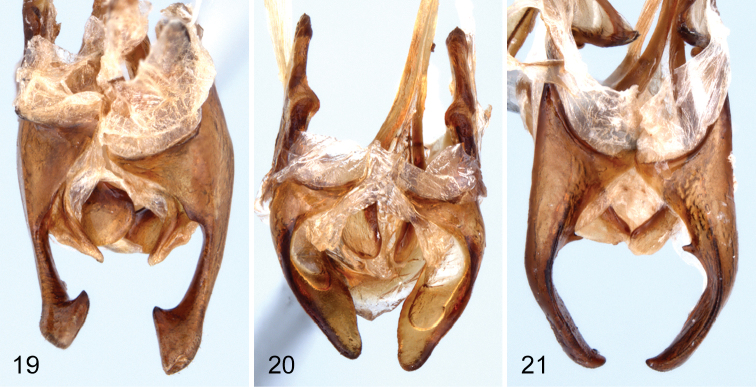
Ventral view of male eighth and ninth ventrites of **19** *Hymenorus balli* **20** *Hymenorus bifurcatus* **21** *Hymenorus excavatus*.

Figs 1 , 4–6 , 13–14 , 19

#### Description.

Body light to dark brown; legs light brown to testaceous; narrowly elongate-oval (Fig. 1). Length 7.5–10.0 mm. Setae short, subrecumbent; uniformly reddish-brown. Eyes moderately separated dorsally (OI of male 19–25 and of female 25–33). Vertex coarsely, densely, punctate; punctures separated by distance approximately half diameter of a puncture. Antennae narrowly elongate, antennomeres 3–11 slightly and evenly widened from base to apex. Apex of sixth antennomere extending posteriad to base of pronotum; antennomeres 3 and 4 subequal in length; tenth antennomere approximately two times longer than wide.

Pronotum distinctly wider than long, PI index 62–71; sides evenly, gradually narrowed from base to apical fourth then evenly curved to continuously curved apical margin; basal angles rectangular; base slightly, but distinctly narrower than base of elytra; basal margin slightly bisinuate; basal foveae small, shallowly impressed, separated by broad, shallow, median impression; midline unimpressed; sides at basal angles slightly reflexed. Disc with fine, dense microsculpture between punctures; punctures coarse, dense, narrowly separated, evenly distributed over disc; each puncture obliquely impressed.

Hypomeron finely, densely, evenly punctate to lateral margins. Basal three abdominal ventrites moderately densely, evenly punctate; punctures each with a short, recumbent seta. Elytra with striae evenly, shallowly impressed; strial punctures circular, almost contiguous along striae; strial interstices slightly convex; interstices moderately densely punctate; punctures randomly distributed, approximately 2 or 3 punctures wide across interval. Metatarsus with basal segment subequal in length to segments 2–4 combined.

Male. Second segment of anterior tarsus without rudimentary lobe on ventral margin. Tibiae not modified. Posterior femora with ventral margin flattened, glabrous, with outer margin of glabrous area distinctly carinate. Anterior tarsal claws each with more than 20 teeth. Metaventrite finely, densely punctate medially; punctures becoming coarser, sparser laterally; median punctures each bearing an elongate, posteriorly directed seta. Fifth abdominal ventrite highly modified (see Figs 4, 5) with large, broad, bifurcate process projecting ventrally from middle of disc; bifurcate process densely setate on outer margins; disc broadly, moderately deeply impressed behind bifurcate process; apical margin of ventrite broadly truncate. Lobes of eighth sternite (Fig. 19) highly modified; narrowed medially with apex broadly widened, inner anterior angle of apical enlargement triangularly narrowed; base of lobes with a moderately long, less heavily sclerotized lobe projecting medially. Lobes of ninth sternite (Fig. 19) short, strongly curved medially, apex broadly rounded. Apicale (Figs 15–16) moderately broad with sides slightly narrowed from base to broadly truncate apex; penis narrowly elongate with sides evenly narrowed from base to apex.

Female. Anterior tarsus without rudimentary lobes or setaceous pads on basal two segments. Anterior tarsal claws each with 9–10 teeth. Metaventrite moderately coarsely punctate medially; punctures becoming slightly coarser, sparser laterally; median punctures each bearing a short, appressed seta. Apical portion of fifth ventrite broadly impressed. Ninth tergite (Fig. 6) with apical margin broadly, evenly convex; with narrow, evenly sclerotized band completely around sides and apex of tergite.

#### Type.

Holotype, male, with labels as follows: “GUAT, 22 km S San Marcos, 5000’, IX-3–1965, JM.Campbell/ HOLOTYPE ♂ Hymenorus balli, desig. 2013, JM Campbell”. The holotype is deposited in the CNCI.

Paratypes. Males 12, females 19.

GUATEMALA: Sacatepequez: Finca Florencia, 24.VI.1993, JMC (CNCI) 1. Quezaltenango: nr. tunnel, 2 km N Santa María, 5500 ft, VII.10.1965, X.24.1965 JMC (JMCC) 3; 3 km N Santa María, 6000 ft, VIII.27.1965, JMC (JMCC) 1. San Marcos: 20 km S San Marcos, 4500 ft, IX.4.1964, JMC (CNCI) 2; 22 km S San Marcos, 5000 ft, IX.3.1965, X.2.1965, JMC (CNCI) 8.

MEXICO: Chiapas: Pico Gallo, NW Union Juarez, 5000–6000 ft, 1972, G. E. Ball (CNCI, JMCC) 16.

#### Etymology.

This species is named in honor of George Ball, University of Alberta, Edmonton in recognition of his many contributions to the study of Coleoptera (particularly Carabidae). Dr. Ball collected a long series of this species from Chiapas, the only record of any of the three species described in this paper from Mexico.

#### Remarks.

The modifications of the male fifth ventrite (Figs 4, 5) are the most unusual of any species of Alleculinae known to me from the New World. In addition to the bizarre modifications of the fifth ventrite, the shape of the male lobes of the eighth and ninth sternites (Fig. 19) and the shape of the apicale (Figs 13, 14) of the aedeagus will readily distinguish the species. The lobe attached to the base of the inner side of each lobe of the eighth sternite is unknown from any other species of the genus. Females are easily distinguished by examination of the eighth tergite (compare Figs 6, 9, 12).

This species was collected by beating arboreal bromeliads and from under moss and bromeliads on tree trunks.

### Hymenorus bifurcatus sp. n.

http://zoobank.org/66308321-E639-48B2-A240-B6F967101AEB

http://species-id.net/wiki/Hymenorus_bifurcatus

Figs 7–9 , 15 , 16 , 20

#### Description.

This species is almost indistinguishable from *Hymenorus balli* and *Hymenorus excavatus* based on non-sexual characters. Only the slight variations in non-sexual characters are described below; full descriptions are given for the male and female characters.

Length 7.9–8.8 mm. Eyes moderately separated dorsally; OI 23 to 27 and of female 28 to 31. PI 72 to 78; basal fovea of pronotum slightly more elongate than in *Hymenorus balli* and *Hymenorus excavatus*, extending from base to near middle of disc. Pronotal punctures angularly impressed throughout.

Metatarsus with basal segment subequal to or slightly longer than segments 2–4 combined.

Male. Anterior tarsus with rudimentary lobe on venter of second segment and pubescent pad on venter of first segment. Posterior femora evenly convex on ventral margin; without carina on outer side of ventral margin. Anterior tarsal claws each with more than 20 teeth (Fig. 3). Fifth ventrite highly modified (see Figs 7, 8), with small, evenly curved process projecting ventrally from middle of disc; apex of process shallowly bifurcate; disc broadly, shallowly impressed behind median process; apical margin broadly convex. Lobes of eighth sternite (Fig. 20) broad, strongly sinuate and curved medially; apex of lobes narrowly rounded; inner sides of lobes broadly, deeply, concavely impressed. Lobes of ninth sternite (Fig. 20) short, narrow, with apical margin moderately narrowly rounded. Apicale (Figs 15, 16) triangular with sides evenly narrowed from base to narrowly rounded apex; penis as in *Hymenorus balli*.

Female. Anterior tarsal claws each with 7–10 teeth. Apical portion of fifth abdominal ventrite narrowly impressed. Ninth tergite (Fig. 9) broad, almost arrowhead shaped with sides widened from truncate apical margin, then abruptly narrowed to base.

#### Types.

Holotype, male, with labels as follows: GUAT, Border of depts. of Sololá and Chimaltenango, nr. Los Robles, IX-12–1965, 6000’, JM Campbell/ HOLOTYPE, ♂ Hymenorus bifurcatus, desig. 2013, JM.Campbell The specimen is deposited in the CNCI.

Paratypes. Males 16, females, 17.

GUATEMALA: Quezaltenango: Tzanjoyan, 3 km SE Zunil, 2300 m, XI.1.1965, JMC (CNCI, JMCC) 5. Sacatepéquez: Finca Florencia, 24.VI.1993, JMC (JMCC) 2. San Marcos: 20 km S San Marcos, 4500 ft, IX.4.1964, JMC (JMCC) 1; 22 km S San Marcos, IX.3.1965, JMC (JMCC) 1. Border between Sololá and Chimaltenango: nr. Los Robles, IX.12.1965, 6000 ft, JMC (CNCI, JMCC) 27.

#### Etymology.

This species is named bifurcatus in recognition of the small, bifurcate process near the middle of the male fifth ventrite.

#### Remarks.

This species is almost identical to the sympatric species *Hymenorus balli* in all external characters except for the lack of a carina on the venter of the posterior femora of the males. The process on the male fifth visible ventrite (Figs 7–8) is somewhat similar to that of *Hymenorus balli* except that it is much smaller and the apical margin of the bifurcate lobes are narrow and shallowly impressed. The lobes of the male eighth sternite (Fig. 20) are very different from those of *Hymenorus balli*; each lobe is broad, spoon-shaped with the apical margin narrowly convex. The deep, concave impression on the inner side of each lobe is unique within the genus. Females may be distinguished by the very different shape of the ninth tergite (compare Figs 6, 9, and 12).

This species was collected by beating dead leaves of recently cut trees, by beating composit shrubs, and from an arboreal bromeliad.

### Hymenorus excavatus sp. n.

http://zoobank.org/268BC875-B177-4E86-A494-2E65EEBC9B3A

http://species-id.net/wiki/Hymenorus_excavatus

Figs 2–3 , 10–12 , 17–18 , 21

#### Description.

This species is almost indistinguishable from *Hymenorus balli* and *Hymenorus bifurcatus* based on non-sexual characters. Only the slight variations in non-sexual characters are described below; full descriptions are given for the male and female characters.

Length 8.0–9.7 mm. Eyes moderately separated (OI of male 18 to 24 and of female 18 to 28).

PI index 66 to 73; sides narrowed from base to apical fourth then evenly curved to slightly concave to truncate anterior margin; median basal fovea more elongate than in *Hymenorus balli*, extending from base to near middle of disc.

Elytra with punctures of intervals slightly denser than in *Hymenorus balli* and *Hymenorus bifurcatus* with 3 or 4 punctures across each interval. Metatarsus with basal segment distinctly longer than segments 2–4 combined.

Male: Venter of anterior tarsi with rudimentary lobes on apex of second segment and densely pubescent pad on basal segment. Posterior femora evenly convex on ventral margin, without carina on outer side. Anterior tarsal claws each with more than 20 teeth. Fifth abdominal ventrite highly modified (Figs 11, 12), broadly, deeply, triangularly impressed from apical margin to basal third; sides of impression sharply carinate. Lobes of eighth sternite (Fig. 21) broadly spoon-shaped, curved medially; outer sides evenly convex, apex of lobes narrowly triangular; inner side of lobes each with short tooth near base. Lobes of ninth sternite (Fig. 21) very short, not extending beyond base of lobes of eighth sternite. Apicale (Figs 17, 18) broad, with sides slightly concave medially; apex truncate; penis narrowly triangular.

Female: Anterior tarsal claws each with 7–10 teeth. Apical third of fifth abdominal ventrite broadly, shallowly, concavely impressed; apical margin broadly convex. Ninth tergite (Fig. 12) with apical margin broadly convex; laterally, slightly truncate medially; heavily sclerotized area covering all of tergite except small, triangular, membranous section medially at base.

#### Types.

Holotype, male, with labels as follows: 22 km S San Marcos, 5000’, IX-3–1965, JM Campbell/ HOLOTYPE ♂, Hymenorus excavatus, desig. 2013, JM.Campbell The specimen is deposited in the CNCI.

Paratypes: 13 males, 11 females.

GUATEMALA: Esquintla: 3 km E San Vicente Pacayá, 5500 ft, V.14.1966, JMC (JMCC) 2. San Marcos: 10 km N La Reforma, 4500 ft, IX.4.1964, JMC (CNCI, JMCC) 7; 22 km S San Marcos, 5000 ft, VI.4.1966, X.2.1965, IX.3.1965, JMC (CNCI, JMCC) 11; 20 km S San Marcos, 4500 ft, IX.4.1964, JMC (JMCC) 2. Suchitepéquez: UVG Reserve, S side of Volcán Atitlán, 91°8.85W, 14°32.04N, X.9–11.2009, 1543 m, (WBWC) 2.

#### Etymology.

This species is named excavatus in recognition of the deeply excavate fifth visible male ventrite.

#### Remarks.

*Hymenorus excavatus* is similar in external appearance to the two preceding species. Males may be readily identified by the unique sexual modifications of the male fifth abdominal ventrite (Figs 10–11), the eighth and ninth sternites (Fig. 21) and the aedeagus (Figs 17–18). Females are difficult to distinguish externally, but can easily be distinguished by the unique shape (somewhat like an arrowhead) of the ninth tergite (compare Figs 6, 9, and 12). Females can be provisionally distinguished from those of *Hymenorus balli* by the shallower and broader impression of the fifth abdomnal ventrite.

## Discussion

These three species are extremely similar to each other except for the very different sexual modifications of the male fifth ventrite and terminalia and the shape of the female ninth tergite (Figs 6, 9, 12). In addition to the unique modifications of the male sexual characters (particularly the modification of the male fifth abdominal ventrite), all three species may be readily distinguished from all other species of *Hymenorus* from southern Mexico and Central America by the combination of their relatively larger size (7.5–10 mm), by having in excess of 20 teeth on the male anterior tarsal claws (Fig. 3), by the more narrowly elongate shape of the body (Figs 1, 2) (most *Hymenorus* species are more broadly elongate). The males of *Hymenorus bifurcatus* and *Hymenorus excavatus* each have a rudimentary tarsal lobe on the second segment and a densely pubescent pad on the venter of the basal tarsal segment of the anterior tarsi (most *Hymenorus* have only the third and fourth anterior tarsal segments lobed ventrally with the basal two segments not modified); the lobes of the eighth sternite of the male of all three species are glabrous (most *Hymenorus* have fine setae on the apex).

A number of species of *Hymenorus* from both North and Central America have an unusual modification of the male posterior femora in which the ventral margin is glabrous, either flattened or concavely impressed with the outer margin distinctly carinate. All of the species having this character also have the male anterior tarsal claws with at least 20 teeth (this character is shared with a few other species having the ventral margin evenly convex and pubescent). Of the three species described as new, *Hymenorus balli* and *Hymenorus excavatus* both have the male posterior femora modified, but *Hymenorus bifurcatus* has the ventral margin evenly convex and pubescent.

The fact that these three species are sympatric in distribution and so similar in non sexual characters and so dissimilar in sexual characters brings to question how and for what selective advantage did these species evolve in such close proximity and what is the purpose of the extreme modifications of the sexual characters?

## Supplementary Material

XML Treatment for Hymenorus

XML Treatment for Hymenorus balli

XML Treatment for Hymenorus bifurcatus

XML Treatment for Hymenorus excavatus
